# Reliability assessment for blood oxygen saturation levels measured with optoacoustic imaging

**DOI:** 10.1117/1.JBO.25.4.046005

**Published:** 2020-04-22

**Authors:** Leonie Ulrich, Kai Gerrit Held, Michael Jaeger, Martin Frenz, Hidayet Günhan Akarçay

**Affiliations:** a University of Bern, Institute of Applied Physics, Biomedical Photonics, Bern, Switzerland; b ABB Switzerland, Corporate Research, Baden-Daettwil, Switzerland

**Keywords:** quantitative optoacoustic imaging, spectral correction, spectral fit, metric, goodness of fit

## Abstract

**Significance:** Quantitative optoacoustic (OA) imaging has the potential to provide blood oxygen saturation (${\mathrm{SO}}_{2}$) estimates due to the proportionality between the measured signal and the blood’s absorption coefficient. However, due to the wavelength-dependent attenuation of light in tissue, a spectral correction of the OA signals is required, and a prime challenge is the validation of both the optical characterization of the tissue and the ${\mathrm{SO}}_{2}$.

**Aim:** We propose to assess the reliability of ${\mathrm{SO}}_{2}$ levels retrieved from spectral fitting by measuring the similarity of OA spectra to the fitted blood absorption spectra.

**Approach:** We introduce a metric that quantifies the trends of blood spectra by assigning a pair of spectral slopes to each spectrum. The applicability of the metric is illustrated with *in vivo* measurements on a human forearm.

**Results:** We show that physiologically sound ${\mathrm{SO}}_{2}$ values do not necessarily imply a successful spectral correction and demonstrate how the metric can be used to distinguish ${\mathrm{SO}}_{2}$ values that are trustworthy from unreliable ones.

**Conclusions:** The metric is independent of the methods used for the OA data acquisition, image reconstruction, and spectral correction, thus it can be readily combined with existing approaches, in order to monitor the accuracy of quantitative OA imaging.

## 1 Introduction

Estimating oxygen levels in blood is of paramount importance in various preclinical and clinical applications, e.g., for the study of tumor characteristics,^1^^,^^2^ personalized cancer treatment,^3^^–^^6^ or the detection and monitoring of cerebral ischemia in newborns.^7^^,^^8^ Quantitative optoacoustic (OA) imaging is an emerging technique that allows the determination of blood oxygen saturation (${\mathrm{SO}}_{2}$) by exploiting the distinct absorption spectra of oxy- and deoxyhemoglobin^9^^,^^10^ while providing a higher spatial resolution in deep tissue than diffuse optical tomography.^11^^,^^12^ The estimation of ${\mathrm{SO}}_{2}$ levels can be achieved by performing spectral fits of blood absorption spectra to measured OA spectra, after correcting them for spectral distortions induced by the wavelength-dependent attenuation of light in tissue.^9^^,^^13^^,^^14^ One major aspect that has recently gained increasing attention is assessing the reliability of the quantitative results.^15^^,^^16^ In this paper, we focus on estimating the uncertainty of the ${\mathrm{SO}}_{2}$ levels retrieved from spectral fitting, independently of the methods used for OA data acquisition, image reconstruction, or for the correction of spectral distortions. More precisely, we dwell on the fact that fitted ${\mathrm{SO}}_{2}$ values that are physiologically reasonable do not necessarily imply that the measured OA spectra follow the trend of real blood spectra. Determining the uncertainty of a fit is a notoriously difficult task that cannot be solved by generally applicable methods.^17^^–^^20^

We propose to assess the trustworthiness of the ${\mathrm{SO}}_{2}$ values by evaluating the similarity between the trends of the measured OA spectra and the trends of the fitted blood spectra. To quantify the spectral trends, we introduce a metric based on the distinctive shape of blood spectra. With the examples of an artery and a vein in a human forearm, we showcase how this metric can be applied to quantitative OA imaging *in vivo*. The measurements were performed using a handheld OA system with a linear array ultrasound (US) transducer, acquiring two-dimensional (2-D) cross-sectional images of the forearm. We start by identifying and analyzing the distorted OA spectra that originate from the vessels of interest, which constitutes an essential part of the quantitative analysis of OA images. For spectral correction, we use multiple irradiation sensing (MIS) that has been described in detail elsewhere.^21^^–^^24^ After spectral correction, we determine the ${\mathrm{SO}}_{2}$ levels in the vessels and assess the reliability of the outcomes using the metric.

## 2 Materials and Methods

### 2.1 Experimental Setup and Data Acquisition

**Fig. 1:**
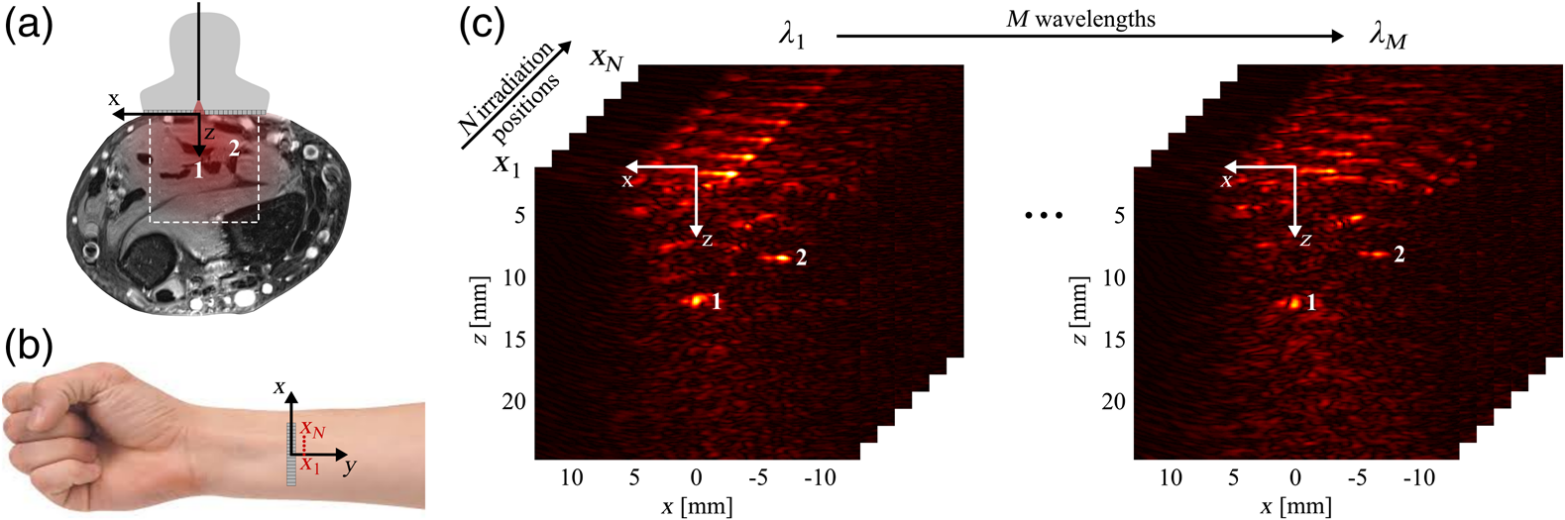
(a) Sketch (not to scale) of the illumination fiber and US probe on a cross-sectional magnetic resonance image of the volunteer’s forearm, displaying the position of the vessels of interest, namely (1) the median artery and (2) a side branch of the median antebrachial vein. (b) Orientation of the linear US transducer array and the irradiation spot, which is translated along x (not to scale). (c) One data set is formed by N·M reconstructed OA images $S_{ij}(r)$, the OA signals of the two vessels of interest are clearly visible.

We performed OA experiments on the inside of the forearm of a healthy volunteer, close to the wrist, to determine the ${\mathrm{SO}}_{2}$ in the median artery located at a depth of z≈12  mm and in a side branch of the median antebrachial vein at z≈8  mm, see Fig. 1(a). The experiments were done in compliance with the ethical principles of the Declaration of Helsinki (2018). The OA system used has been described in detail in Ref. 24. In short, we illuminated the tissue using a diode-pumped Q-switched Nd:YAG laser (Spitlight DPSS OPO, InnoLas Laser GmbH, Germany) with integrated optical parametric oscillator. The light was coupled into a multimode fiber (Thorlabs), which was fixed on a motorized translation stage (T-series, Zaber, Canada), allowing an automated stepwise translation of the irradiation spot. For OA signal detection, we employed a linear array US probe (ATL L7-4, Philips N.V., The Netherlands) connected to a research US system (V1-64, Verasonics). The transducer was placed on the forearm, the linear array being oriented perpendicularly to the longitudinal axis y of the arm, defining y=0 [see Figs. 1(a) and 1(b)]. The arm was immobilized by an arm holder to reduce motion artifacts.^25^ US gel was used for acoustic coupling between the transducer and the skin. While acquiring real-time OA and US images, the position of the transducer was adjusted such that the vessels of interest became visible in the field of view of the transducer. The initial x position of the tip of the illumination fiber, $x_{1}$, was chosen to be directly above the median artery, i.e., to correspond to the minimal distance between the irradiation spot on the skin and the artery. The y position of the fiber tip was kept at $y_{0}=19\;\mathrm{mm}$. To perform an MIS acquisition sequence (see Supplementary Material), the fiber tip was translated along the x axis with a step size of 0.5 mm, over a total distance of 9.5 mm, resulting in N=20 different irradiation spot positions $x_{i}$, i∈{1,…,N} [see Fig. 1(b)]. At each position, 150 OA acquisitions were averaged to increase the signal-to-noise ratio (SNR). The surface of the arm was flat within the range scanned with the irradiation spot position, and the position $x_{N}$ of the outermost irradiation spot was around 20 mm away from the edge of the arm (top-down view). Therefore, the experimental conditions roughly complied with the assumptions made by the MIS approach with regard to tissue boundaries. The MIS acquisition sequence was repeated for M=7 wavelengths $\lambda_{j}$, j∈{1,…,N} (see Table 1). The number of wavelengths was chosen as a trade-off between the robustness of the spectral fit and an acceptable acquisition time. Although, in general, a higher number of wavelengths would be desirable, the resulting increase in acquisition time would at the same time have led to increased motion artifacts. The set of wavelengths was chosen so that it contained the isosbestic point at $\lambda_{\mathrm{iso}}\approx 800\;\mathrm{nm}$ (needed for the reliability assessment, see Sec. 2.3), and an equal number (3) of wavelengths left and right of $\lambda_{\mathrm{iso}}$. To the right of $\lambda_{\mathrm{iso}}$, the wavelength range was limited by the stability of the laser as well as by a low SNR. To the left, we distributed the three wavelengths to capture the spectral peak around λ=760  nm for deoxygenated blood. Overall, we sampled the range between λ=740  nm and λ=890  nm with wavelengths spaced equidistantly within the wavelength intervals to the left and right of the isosbestic point. A frequency-domain algorithm^26^ was employed to reconstruct N·M radio-frequency (rf-)mode images with a pixel resolution of 149  μm×77  μm in the x and z direction, respectively. Further analysis was performed based on the envelopes of these rf-mode images, which will from now on be referred to as OA images and denoted by $S_{ij}(r)$, for irradiation positions $x_{i}$ and wavelengths $\lambda_{j}$. The signals stemming from the artery and the vein are clearly visible in the OA images [see Fig. 1(c)], in the following they will be called OA signals. To be able to correct for wavelength-dependent variations in laser output, the average pulse energies were recorded for each wavelength $\lambda_{j}$. During the OA measurements, a standard pulse oximeter (Nellcor Oximax NPB-40, Medtronic, Ireland) was used to measure the reference arterial ${\mathrm{SO}}_{2}$ of the volunteer.

**Table 1:** Wavelengths used for OA acquisitions.

j	1	2	3	4	5	6	7
$\lambda_{j}$ (nm)	740	760	780	800	830	860	890

### 2.2 Motion Correction, Identification of Support, and Pixelwise SO_2_ Determination

**Fig. 2:**
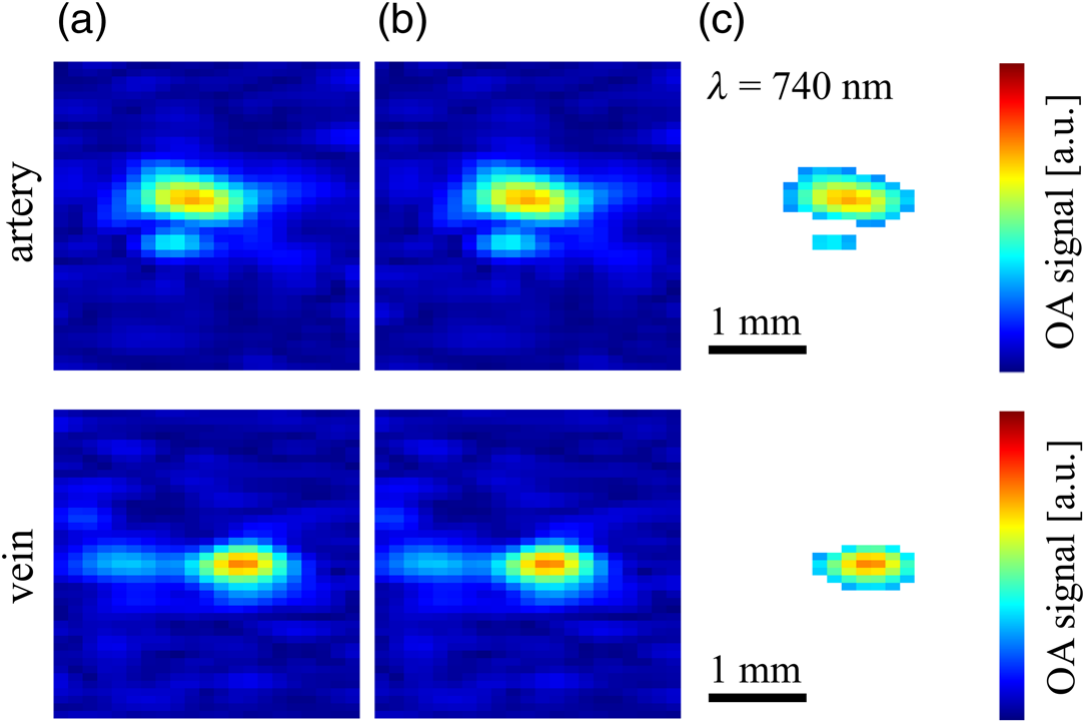
OA signals emanating from (top) the artery and (bottom) the vein for the different wavelengths $\lambda_{j}$, for one irradiation position, (a) before and (b) after motion correction, together with (c) segmented supports (Video 1, MPEG, 4.5 MB [URL: https://doi.org/10.1117/1.JBO.25.4.046005.1]).

Figure 2(a) shows the OA signals recorded from both the artery and the vein, for one irradiation position. Part (a) in the animation (Video 1) reveals that, despite the use of an arm holder for immobilization, the vessel positions fluctuate between different $\lambda_{j}$. To compensate for these fluctuations, we defined the x and z positions of the vessels, $\left(x_{ij}^{c},z_{ij}^{c}\right)$, by manually identifying the centers of the corresponding OA signals in all N·M OA images and subsequently shifted the images such that all $\left(x_{ij}^{c},z_{ij}^{c}\right)$ coincide. Part (b) of the animation (Video 1) displays the OA signals after motion correction and confirms that the centers are congruent. However, due to noise and out-of-plane motion, the size, shape, and 2-D profile of the OA signals vary with wavelength, introducing an uncertainty in the analysis of the OA signals. To account for this uncertainty, we perform, for each OA signal, a statistical analysis over the pixels corresponding to the respective vessel. These pixels were identified by employing a simple thresholding method (such as, e.g., in Refs. 27–29), assuming that the intensity of signals originating from vessels significantly exceeds the signal intensity in the background. As a threshold, we chose the pixel intensity that corresponded to 1/e of the maximum pixel value within a manually selected region of interest covering the vessel’s OA signal. In consistence with the terminology introduced in Ref. 12, we call the selected set of pixels the support, defined as “a set of pixels in which the signal values … are determined by the optical absorption inside … [the] vessel”. Figure 2(c) shows the supports for both the artery and the vein, for the same OA signals that were presented in Figs. 2(a) and 2(b). Separately for each irradiation position $x_{i}$, the pixelwise analysis of the spectra was performed on the subset of pixels that were part of the support for all M=7 wavelengths. The decision to exclude pixels from the analysis was motivated by the following rationale: on the one hand, we did not want to include pixels that—for one or more wavelengths—did not fulfill the criterion for the support, as such pixels were not considered to reliably reflect the absorption of the blood in the vessel. On the other hand, we did not want to include pixels with less than M=7 spectral data points, as 7 is already a very small number with regard to the spectral analysis.

For the pixels that were part of the support for all wavelengths, we performed the analysis of the OA spectra both before and after correction of distortions induced by the wavelength-dependent attenuation of light in the tissue. As mentioned earlier, the spectral correction was achieved by optically characterizing the forearm tissue according to the MIS principle (details on the spectral correction technique are given in the Supplementary Material and in Video S1, MPEG, 5.3 MB [URL: https://doi.org/10.1117/1.JBO.25.4.046005.4] and Video S2, MPEG, 5.6 MB [URL: https://doi.org/10.1117/1.JBO.25.4.046005.5]). We determined the effective attenuation coefficient $\mu_{\mathrm{eff}}$ of the tissue for every wavelength $\lambda_{j}$ and analytically calculated the fluences $\Phi_{ij}(r)$ in the OA image plane for $x_{i}$ and $\lambda_{j}$, with the pixel resolution of the OA images. The spectral correction is then simply a pixelwise division of OA images $S_{ij}(r)$ by $\Phi_{ij}(r)$. Subsequently, ${\mathrm{SO}}_{2}$ levels were determined by spectral fitting to the corrected OA spectra, for every individual pixel in the supports.

### 2.3 Metric for Quantitative Characterization of Spectral Trends

**Fig. 3:**
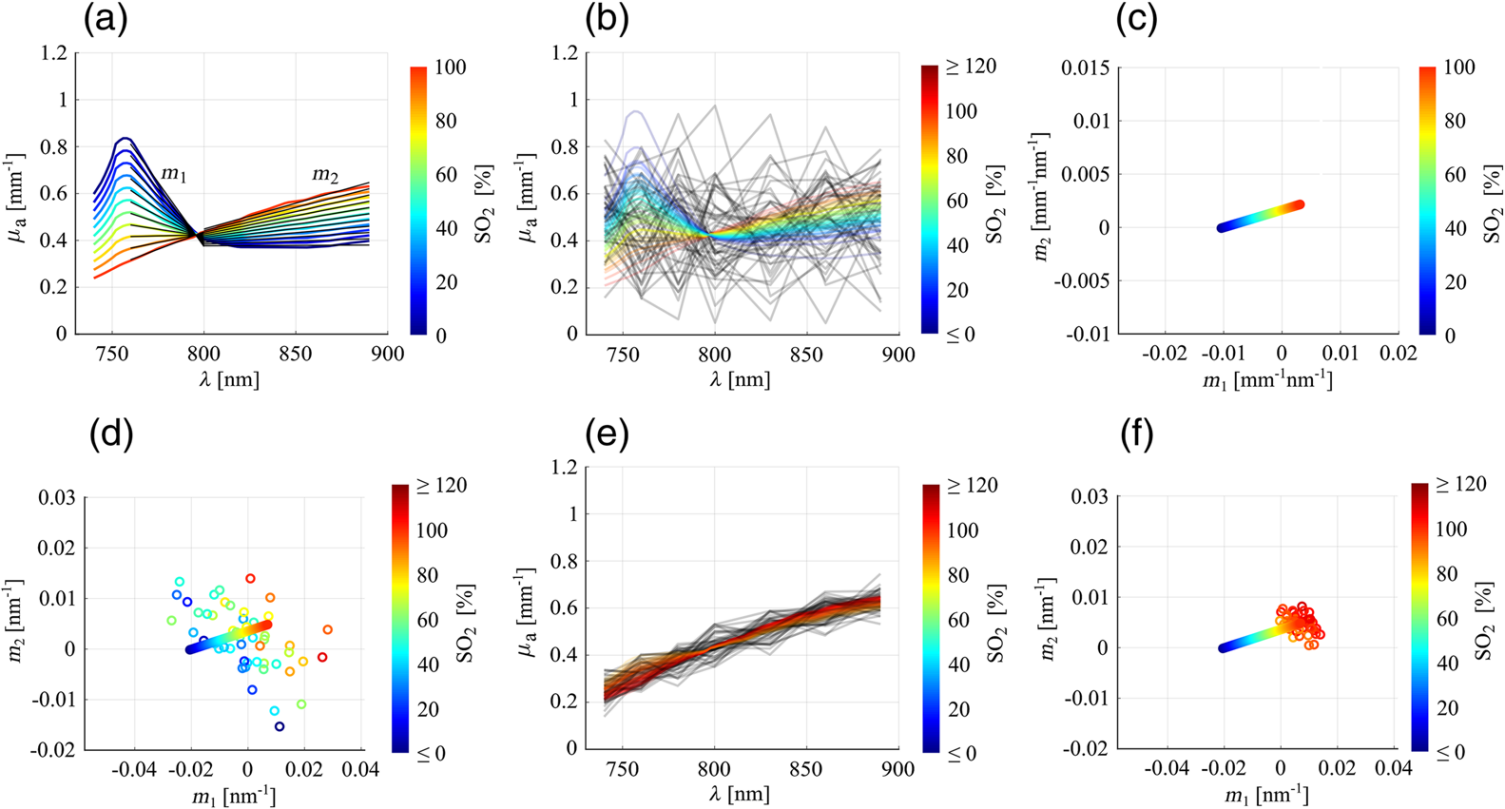
(a) Blood spectra for ${\mathrm{SO}}_{2}$ levels between 0% (blue) and 100% (red), together with the fitted first-order polynomials (in black) to the spectra left and right of the isosbestic point. (b) Synthetic OA spectra (random Gaussian noise, in black) with fitted blood spectra (in color), colors indicating the respective ${\mathrm{SO}}_{2}$ levels. (c) Pairs of the two slopes $m_{1}$ and $m_{2}$ determined for the blood spectra shown in (a), constituting the reference line for the metric plots. (d) Metric plot for the synthetic OA spectra shown in (b): reference line together with slope pairs ($m_{1}$, $m_{2}$) determined for the synthetic OA spectra (circles), colors indicate the ${\mathrm{SO}}_{2}$. (e) Synthetic arterial blood spectra (in black), generated by adding random Gaussian noise to the absorption spectrum of blood at ${\mathrm{SO}}_{2}=98\%$, together with fitted spectra (in color), colors indicating the respective ${\mathrm{SO}}_{2}$ levels. (f) Metric plot for the spectra shown in (e): reference line together with slope pairs ($m_{1}$, $m_{2}$) determined for the synthetic OA spectra (circles), colors indicate the ${\mathrm{SO}}_{2}$. For the visualization of the OA spectra in (b) and (e), each OA spectrum was divided by the scaling factor A given by the respective spectral fit. Further note that the change in units between (c) and (d), (f) is caused by the normalization of the spectra before determining ($m_{1}$, $m_{2}$); details are given in the text.

In this section, we address the central issue of this paper, namely the importance of assessing the reliability of the spectral fits. In Fig. 3(a), we have plotted blood absorption spectra in the wavelength range used for the experiments, for ${\mathrm{SO}}_{2}$ values between 0% and 100% with a step size of 10%, the respective ${\mathrm{SO}}_{2}$ level being indicated in color code. To construct these spectra, we extracted the absorption spectra $\mu_{a}^{{\mathrm{HbO}}_{2}}$ and $\mu_{a}^{\mathrm{Hb}}$ of oxy- and deoxyhemoglobin from Ref. 30 and, assuming that hemoglobin is the dominant absorber in blood,^31^ calculated the absorption spectrum of blood at a particular ${\mathrm{SO}}_{2}$ as the respective linear combination of $\mu_{a}^{{\mathrm{HbO}}_{2}}$ and $\mu_{a}^{\mathrm{Hb}}$. It can be seen that the blood spectra in Fig. 3(a) follow a range of trends. This range is such that a spectral fit can result in an ${\mathrm{SO}}_{2}$ value that can be interpreted as physiologically reasonable, even if the measured OA spectrum is not an actual blood spectrum. We illustrate such a situation in Fig. 3(b), where we have generated synthetic OA spectra reflecting random Gaussian noise (black lines, the number of spectra roughly corresponds to the number of pixels we found in one support in this study), to which we fitted blood absorption spectra (colored lines, where the color indicates the fitted ${\mathrm{SO}}_{2}$ parameter). For the spectral fit, we determined the blood absorption spectrum in the wavelength range between λ=740  nm and λ=890  nm that matches the OA data best in the least-squares sense, using the fit function $y(\lambda )=A\cdot [\mu_{a}^{{\mathrm{HbO}}_{2}}(\lambda )\cdot ({\mathrm{SO}}_{2}/100)+\mu_{a}^{\mathrm{Hb}}(\lambda )\cdot (1-{\mathrm{SO}}_{2}/100)]$ with the ${\mathrm{SO}}_{2}$ (in %) and a scaling factor A as fit parameters. Note that the factor A accounts for the fact that the absolute amplitude of a real OA signal detected with a linear array probe does not bear a quantitative meaning as it depends via the system impulse response on the vessel orientation,^32^ which induces an unknown scaling of OA spectra. Even though the OA spectra shown in Fig. 3(b) are random noise, the corresponding spectral fits result in ${\mathrm{SO}}_{2}$ values mostly between 0% and 100%. This highlights that the mere fact that a fit yields a physiologically possible ${\mathrm{SO}}_{2}$ value does not necessarily imply that the OA spectrum is well described by the fitted blood spectrum and the ${\mathrm{SO}}_{2}$ value is meaningful.

Assessing the reliability of the outcome of a fit is known to be a nontrivial task, which often necessitates *ad hoc* solutions.^19^^,^^33^ Standard goodness-of-fit parameters, like, e.g., $\chi^{2}$ (the sum of squared residuals) or $R^{2}$, have limited suitability for our purpose because they cannot identify whether the differences between the data points and the fitted curve are biased (i.e., systematic). On the other hand, analyzing the quality of the spectral fit using more sophisticated statistical procedures is usually impeded by a very low number of available data points (wavelengths) as a consequence of limits in the acquisition time acceptable for *in vivo* measurements, together with technical constraints of the OA experiment. Instead, we suggest to use a customized method that is tailored to the analysis of fits of blood spectra and easy to use. More specifically, we propose to determine the similarity between the trend of the measured OA spectrum and the trend of the fitted blood spectrum.

To quantify the trends of blood spectra, we exploit a distinctive feature in the wavelength range between λ=760  nm and λ=890  nm, namely that the trends of the spectra before and after the isosbestic point at around $\lambda_{\mathrm{iso}}\approx 800\;\mathrm{nm}$ can be approximated by straight lines with slopes $m_{1}$ and $m_{2}$, respectively [see black lines in Fig. 3(a)]. We use the slope pair ($m_{1}$, $m_{2}$) as a metric and determine the values of $m_{1}$ and $m_{2}$ by performing least-squares fits of two first-order polynomials to the spectra on the left and right sides of the isosbestic point, respectively. While the spectral fit to determine the ${\mathrm{SO}}_{2}$ was performed including the full wavelength range between λ=740  nm and λ=890  nm, the polynomial fits to determine the slopes $m_{1}$ and $m_{2}$ were performed on the two subsets of wavelengths {760  nm,780  nm,800  nm} and {800  nm,830  nm,860  nm,890  nm}, respectively. λ=740  nm was not included for the polynomial fits as this wavelength is outside the range in which blood spectra are approximately linear, see Fig. 3(a). The pairs ($m_{1}$, $m_{2}$) corresponding to actual blood with $0\%\leq {\mathrm{SO}}_{2}\leq 100\%$ constitute a reference and are visualized in a scatter plot [$m_{2}$ against $m_{1}$, see Fig. 3(c)] as color-filled points, where the color denotes the corresponding ${\mathrm{SO}}_{2}$ value, like in Fig. 3(a). It can be seen that the points form a line, in the following called reference line. Likewise, to quantify the trend of a measured OA spectrum, we determine the slope pair ($m_{1}$, $m_{2}$) by performing least-squares fits of two first-order polynomials to the OA data points left and right of the isosbestic point. Due to the aforementioned unknown scaling of the measured OA spectra, direct fits to the OA spectra would not result in slopes that are comparable to those of actual blood. To obtain ($m_{1}$, $m_{2}$) values that do not depend on the scaling, we normalized the OA spectra before performing the polynomial fits to determine ($m_{1}$, $m_{2}$). We normalized each OA spectrum by dividing it by its respective mean value across the full wavelength range between λ=740  nm and λ=890  nm. The same normalization was used for a redefinition of the reference line to ensure comparability, i.e., we divided each blood spectrum by the respective mean value over the set of wavelengths used for the OA measurement, before determining the reference slope pairs ($m_{1}$, $m_{2}$). Note that this normalization causes a change in magnitude and unit of the reference ($m_{1}$, $m_{2}$) with respect to the original reference line shown in Fig. 3(c). To measure the similarity between the trend of a measured OA spectrum and the trend of the blood spectrum determined in the corresponding spectral fit, the slope pair ($m_{1}$, $m_{2}$) for the OA spectrum is visualized as a colored circle in the scatter plot together with the reference line (the color of the circle designating the ${\mathrm{SO}}_{2}$ level given by the spectral fit). The resulting scatter plot containing the reference line and the circles for all OA spectra in the support, referred to as metric plot, allows one to assess the reliability of the spectral fits.

We illustrate this with two examples. In the first example in Fig. 3(d), the position of each circle represents the pair ($m_{1}$, $m_{2}$) for one of the synthetic OA spectra given in Fig. 3(b). The positions of the circles mostly far away from the reference line indicate that no systematic similarity can be established between the trends of the OA spectra and those of the fitted spectra, meaning that the fits, even though they may result in physiologically possible ${\mathrm{SO}}_{2}$ values, are not trustworthy. In case of a high similarity between the trends of measured OA spectra and the trends of fitted blood spectra, each colored circle would be close to the respective point in the reference line having the same color. Such a situation is illustrated with a second example, see Figs. 3(e) and 3(f). Figure 3(e) shows synthetic OA spectra (black lines) for an artery, numerically generated by adding random Gaussian noise to the absorption spectrum of blood at ${\mathrm{SO}}_{2}=98\%$ (which is a typical ${\mathrm{SO}}_{2}$ level for arterial blood^34^). The SNR was adjusted to be comparable to that observed in our OA images. Again, the number of spectra was chosen to roughly correspond to the number of pixels we obtained in a support. The red color of the fitted blood spectra indicates that the reference ${\mathrm{SO}}_{2}$ (98%) has been retrieved correctly. The corresponding metric plot is given in Fig. 3(f). Two main observations can be made. First, the circles describing the trends of the OA spectra across the support are clustered, which contrasts with the situation given in Fig. 3(d). This means that we can identify a systematic trend, i.e., the trends of OA spectra do not differ much between individual pixels, despite the stochastic fluctuations in the spectra. Note that a statistically meaningful number of spectra, provided by the pixelwise analysis in a support, is a prerequisite for the identification of a cluster in the corresponding metric plot. Second, the position of the cluster agrees with the position of the points on the reference line covering the same color range. These two observations together indicate that the trends of the OA spectra do indeed systematically resemble the trends of the fitted blood spectra. The metric plot combines all the relevant information needed to comprehensively represent the outcomes of an ${\mathrm{SO}}_{2}$ estimation, namely (i) information on the uniformity of the spectral trends across the support (clustering of circles), (ii) the fitted ${\mathrm{SO}}_{2}$ values across the support (colors of circles), and (iii) information on the similarity between the OA spectra and the fitted blood spectra (proximity of circles to points on the reference line covering the same color range).

## 3 Results and Discussion

### 3.1 Quantitative Analysis of Uncorrected OA Spectra

In a first step, we would like to demonstrate based on our *in vivo* data that the metric can already be used to quantitatively analyze the measured OA spectra before correction. The analysis of uncorrected spectra can be helpful as, in case of an unsuccessful spectral correction, it would be difficult to distinguish potential sources of error related to the different steps involved in the analysis if merely the corrected OA spectra were investigated. In particular, applying a spectral correction and determining ${\mathrm{SO}}_{2}$ levels is only sensible if one is certain that the corresponding supports have been correctly identified and that motion artifacts have been corrected for. We show that this can be ascertained using parts of the information given in the metric plots, namely by observing the clustering of slope pairs.

**Fig. 4:**
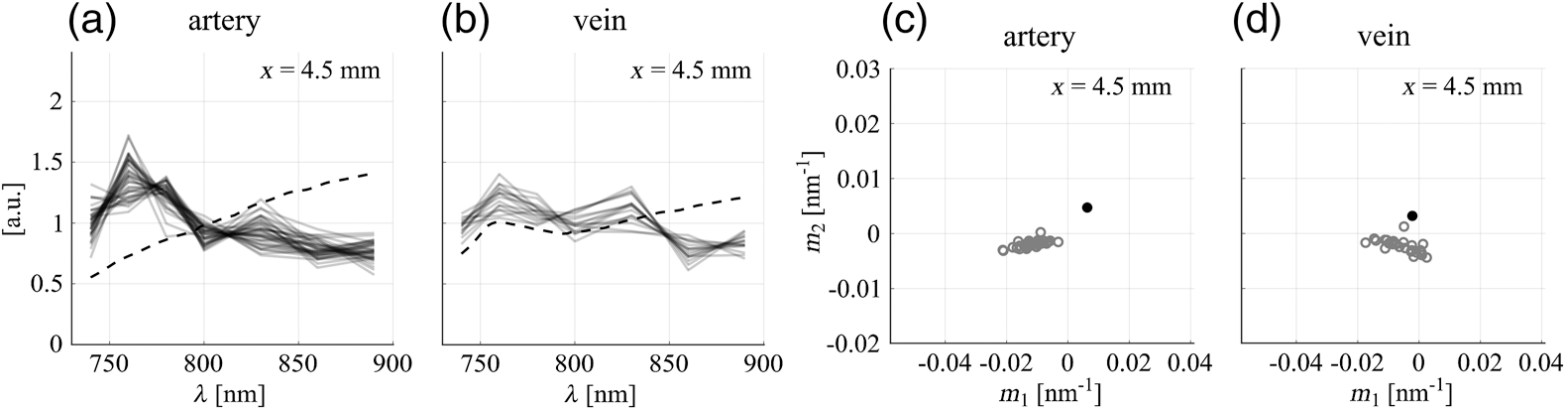
(a), (b) Uncorrected OA spectra corresponding to individual pixels within the support (black lines), for the artery and the vein, respectively. Reference blood spectra are shown as dashed lines and correspond to the reference ${\mathrm{SO}}_{2}$ level determined by pulse oximetry (${\mathrm{SO}}_{2}=98\%$) and an average literature value (${\mathrm{SO}}_{2}=70\%$), respectively. For the visualization, each uncorrected OA spectrum and reference blood spectrum was normalized by dividing it by its respective mean value over the set of wavelengths used for the OA measurement. (c), (d) Reduced metric plots for spectra shown in (a), (b), pairs ($m_{1}$, $m_{2}$) determined for the OA spectra are shown as gray circles, ($m_{1}$, $m_{2}$) for the reference blood spectra are given as black points. A video showing the figure for all $x_{i}$ is available (Video 2, MPEG, 8.4 MB [URL: https://doi.org/10.1117/1.JBO.25.4.046005.2]).

Figures 4(a) and 4(b) show the uncorrected OA spectra (black lines) for all pixels within the supports for the artery and the vein, respectively, for one irradiation position. According to the definition of the support (recall Sec. 2.2), every pixel in the support is assumed to reflect the wavelength dependency of the total energy absorbed in the corresponding vessel and thus the OA spectra are expected to be uniform across the support. This assumption is reasonable if either (i) the optical penetration depth is much larger and/or (ii) the vessel diameter is much smaller than the size of the point spread function of the imaging system.^35^ In this study, the first condition is roughly fulfilled [the size of the point spread function is ∼0.5  mm and the penetration depth is around 2 mm ($\mu_{a}\approx 0.5\;{\mathrm{mm}}^{-1}$)] and, in agreement with the expectation, the spectra corresponding to individual pixels of one support are relatively uniform, both for the artery and the vein. The small variations observed between individual spectra are a result of differences in the size, shape, and 2-D profile of the supports that occurred between the acquisitions of data sets corresponding to different wavelengths (recall Sec. 2.2). The variations are slightly larger for the vein due to a lower SNR. Nevertheless, for both the artery and the vein, it can be seen that the spectra follow a systematic trend.

Furthermore, it is apparent in Figs. 4(a) and 4(b) that the trends of the spectra recorded from the artery and the vein are distorted with respect to the actual absorption spectra of the blood in the vessels (dashed lines), due to the wavelength-dependent attenuation of the light. The reference spectra correspond to ${\mathrm{SO}}_{2}=98\%$ (given by the pulse oximeter measurement) for the artery and to ${\mathrm{SO}}_{2}=70\%$ (average literature value^36^^–^^38^) for the vein. Interestingly, the OA spectra are very similar to blood spectra at low ${\mathrm{SO}}_{2}$ levels [compare Fig. 3(a)]. However, this similarity is not relevant as for vessels located at these depths in the tissue, the spectral distortion is generally expected to be significant and thus uncorrected OA spectra most likely do not represent the actual absorption spectra of the blood in the vessels. In fact, spectral fits to the uncorrected OA spectra shown in Figs. 4(a) and 4(b) result in unphysiologically low ${\mathrm{SO}}_{2}$ values, i.e., around 20% for the artery and 30% for the vein.

The qualitative observations in Figs. 4(a) and 4(b) are summarized in a condensed way in a reduced representation of the metric plot. Since, as mentioned above, before correction there is generally no reason to expect that the trends of the OA spectra follow the ones of actual blood, a comparison between the positions of the slope pairs and the reference line [as it was done in Figs. 3(d) and 3(f)] is not meaningful. Hence, the reduced metric plot does not include information on fitted ${\mathrm{SO}}_{2}$ levels and omits the reference line. In Figs. 4(c) and 4(d), the reduced metric plots are given for the spectra shown in Figs. 4(a) and 4(b), respectively. The existence of systematic trends in the measured OA spectra is underlined by a strong clustering of slope pairs ($m_{1}$, $m_{2}$) both for the artery and the vein, corroborating the good performance of the motion correction and segmentation procedure outlined in Sec. 2.2 and thus confirming that it is sensible to proceed to the spectral correction.

In addition, the reduced metric plots allow one to visualize the distortion of the OA spectra with respect to the reference blood spectra for the artery and the vein. For this purpose, we have added the reference slope pairs (corresponding to ${\mathrm{SO}}_{2}=98\%$ and ${\mathrm{SO}}_{2}=70\%$, respectively, displayed as black points). A measure for the spectral distortion is given by the position of the cluster with respect to the position of the reference point: for the artery, the distance between the cluster and the reference point is larger than for the vein, confirming the stronger distortion of the spectra in Figs. 4(a) than in 4(b). The degree of distortion of measured OA spectra is generally expected to depend on the distance between the irradiation spot at the tissue surface and the blood vessel. According to Eq. (1) in the Supplementary Material, given a particular wavelength dependency of $\mu_{\mathrm{eff}}$, a larger distance from the illumination source leads to more pronounced differences in the fluence between different wavelengths and thus to a more pronounced spectral distortion. Although the artery is located at a larger depth than the vein, the distance between the irradiation spot and the vessel is, for the irradiation position x=4.5  mm, larger for the vein than for the artery, due to the irradiation geometry used in the experiment. Therefore, the stronger distortion for the artery cannot be explained by the distance from the illumination source. A plausible reason for the observed differences in the distortion of the OA spectra could be differences in the optical properties of the two tissue segments that are located between the skin and the artery/vein.

### 3.2 Quantitative Analysis of Corrected OA Spectra

After having established the good performance of the motion correction and segmentation procedure, we proceed by showing the results of the spectral correction.

**Fig. 5:**
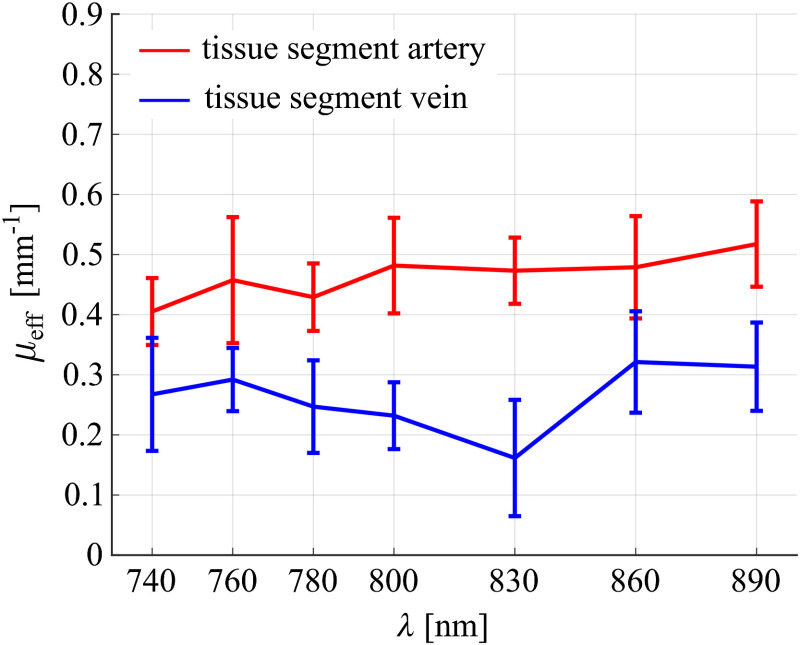
$\mu_{\mathrm{eff}}$ spectra estimated for the tissue between the irradiation points at the forearm surface and the artery (red) and the vein (blue).

Figure 5 displays the spectra of the effective attenuation coefficient ($\mu_{\mathrm{eff}}$) used for the correction of the OA spectra. According to the MIS approach, the $\mu_{\mathrm{eff}}$ spectrum assigned to each blood vessel is expected to optically characterize a tissue segment for which the diffusion approximation is valid (see Supplementary Material). Since these two tissue segments differ (they are defined by different subsets of irradiation positions at the forearm surface and different positions of the vessels, see Supplementary Material), they might have different optical properties. This is a possible explanation for the significant difference between the $\mu_{\mathrm{eff}}$ obtained for the artery and the vein. For both tissue segments, the $\mu_{\mathrm{eff}}$ spectra are in line with the broad range of effective attenuation coefficients of human tissue reported in the literature (approximately between 0.1 and $1\;{\mathrm{mm}}^{-1}$).^39^^–^^43^ Note that, for both the artery and the vein, spectral correction was only performed for the respective subset of irradiation positions corresponding to the tissue segment that is optically characterized by the $\mu_{\mathrm{eff}}$ given in Fig. 5.

**Fig. 6:**
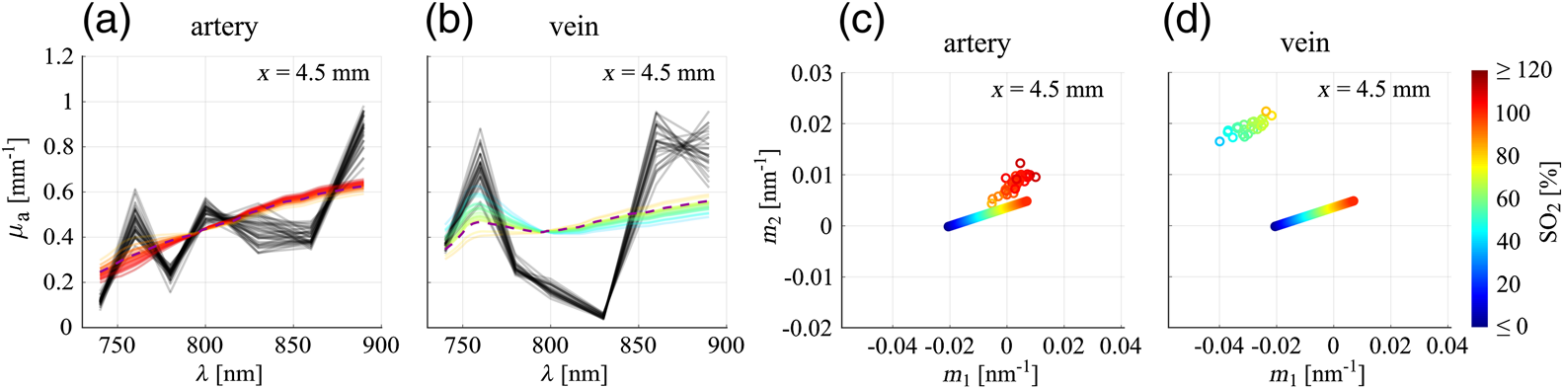
(a), (b) Corrected OA spectra corresponding to individual pixels within the support (black lines), for the artery and the vein, respectively, together with the fitted spectra that are plotted in color to indicate the respective ${\mathrm{SO}}_{2}$ levels. For the visualization, each OA spectrum was divided by the scaling factor A given by the respective spectral fit. Reference spectra are shown as purple dashed lines and correspond to the reference ${\mathrm{SO}}_{2}$ level determined by pulse oximetry (${\mathrm{SO}}_{2}=98\%$) and an average literature value (${\mathrm{SO}}_{2}=70\%$), respectively. (c), (d) Metric plots for spectra shown in (a), (b), representing the reference line together with the pairs ($m_{1}$, $m_{2}$) determined for the corrected OA spectra. A video showing the figure for all $x_{i}$ is available (Video 3, MPEG, 7.7 MB [URL: https://doi.org/10.1117/1.JBO.25.4.046005.3]).

The OA spectra retrieved after correction are shown as black lines in Figs. 6(a) and 6(b), for the artery and the vein, respectively, together with the blood spectra determined by the spectral fits. It can be clearly seen that the corrected OA spectra exhibit strong fluctuations across the wavelength range. It is reasonable to assume that parts of these fluctuations can be explained by errors in $\mu_{\mathrm{eff}}$: it can be observed, by looking at Fig. 5, that fluctuations in the $\mu_{\mathrm{eff}}$ spectra, although they are less pronounced, are very similar to the fluctuations in the corrected OA spectra. This observation is plausible if one assumes that the observed fluctuations in $\mu_{\mathrm{eff}}$ are due to measurement errors relative to true $\mu_{\mathrm{eff}}$ spectra that are much smoother than the measured $\mu_{\mathrm{eff}}$ spectra. Such errors would indeed appear in the corrected OA spectra, because, as explained in Sec. 2.2, $\mu_{\mathrm{eff}}$ is used to calculate the fluence $\Phi_{ij}(r)$, and the corrected OA spectra are determined by performing a division of the OA image $S_{ij}(r)$ by the fluence $\Phi_{ij}(r)$. Due to the exponential dependence of the fluence on $\mu_{\mathrm{eff}}$ [see Eq. (1) in the Supplementary Material], errors in the $\mu_{\mathrm{eff}}$ spectra appear in an amplified way in the corrected OA spectra.

As mentioned earlier, the goal of this paper is to demonstrate the necessity of assessing the reliability of spectral fits. Qualitatively, it can be seen in Figs. 6(a) and 6(b) that, for the vein, the level of fluctuations in the OA spectra around the fitted blood spectra is larger than for the artery. This can already be regarded as an indication of a difference in the reliability of the spectral correction between the artery and the vein, and one could, e.g., attempt to quantify the reliability based on $\chi^{2}$, which would represent a measure for the level of fluctuations in the OA spectra. With regard to the reliability assessment of the ${\mathrm{SO}}_{2}$ estimation, however, the level of fluctuations is not the main deciding factor. We argue that the more fundamental question is to what extent the corrected OA spectra have a systematic trend that resembles that of actual blood. As already explained in Sec. 2.3, simply fitting blood spectra to the corrected OA spectra can be misleading: the ${\mathrm{SO}}_{2}$ values corresponding to the fitted blood spectra in Figs. 6(a) and 6(b) (indicated in color) are physiologically realistic both for the artery (fitted ${\mathrm{SO}}_{2}$ values around 100%) and the vein (fitted ${\mathrm{SO}}_{2}$ values around 60%). For the artery, it can be seen that the OA spectra fluctuate around the monotonically increasing blood spectra determined by the spectral fits, whereas a substantial deviation in the trends between OA spectra and fitted spectra can be observed for the vein. To quantify this qualitative observation, we use the metric introduced in Sec. 2.3, as it provides a measure to quantify the agreement between spectral trends that is—as we argue—more relevant for the ${\mathrm{SO}}_{2}$. The metric plots corresponding to the corrected OA spectra shown in Figs. 6(a) and 6(b) are given in Figs. 6(c) and 6(d), respectively. For the artery, the colored circles are clustered close to the filled points on the reference line covering the same color range. This underlines that the corrected OA spectra systematically follow the trends of the fitted blood spectra [both observations stated in Sec. 2.3 are fulfilled, like in the example shown in Fig. 3(f)] and attests the trustworthiness of the fits (note here that we interpret the cluster’s distance from the reference line in relation to its spread: for the artery, the distances of the circles from the reference line are smaller than or comparable to the extent of the cluster). Moreover, the proximity of the cluster to the point on the reference line corresponding to ${\mathrm{SO}}_{2}=98\%$ indicates a reasonable spectral correction, as the cluster is located closer to the reference point than before correction. For the vein, the circles are also clustered, however, their positions are way off the positions of the points on the reference line covering the same color range, which is a clear indication that the fits are not reliable and that the fitted ${\mathrm{SO}}_{2}$ levels, although they are physiologically realistic, are not to be trusted. Here, the erroneousness of the spectral correction is *de facto* proven without the need of a reference ${\mathrm{SO}}_{2}$, as the distance between the cluster and the reference line in the metric plot reveals the dissimilarity between the corrected OA spectra and actual blood spectra.

Based on these metric plots, it is also possible to quantify a cluster’s distance from the reference line in relation to its spread, in order to provide a number that indicates the similarity between the trends of the corrected OA spectra and the trends of the fitted blood spectra. A possible approach to measure the distance of the cluster from the reference line is by determining the mean value of the distances of individual circles in the cluster from the point on the reference line that corresponds to the average ${\mathrm{SO}}_{2}$ in the cluster. An approximate measure for the spread of the cluster is, e.g., given by the mean distance of the individual circles from the centroid of the cluster. In the ideal case where the cluster’s centroid coincides with the reference point, the ratio between these two quantities would be 1. In this study, we obtain a ratio of 1.71±0.31 for the artery (mean and standard deviation over all irradiation positions) and a ratio of 7.84±3.58 for the vein. The ratio obtained for the artery is thus close to the ideal case, underlining the accuracy of the correction of the artery’s OA spectra, whereas the one obtained for the vein is much larger. With regard to a clinical application, one could define a threshold for the aforementioned ratio to identify unreliable results. However, the definition of such a threshold would require more statistics than we can provide, depends on the clinical target, and was therefore beyond the scope of this paper.

From the observed dissimilarity between the corrected OA spectra and actual blood spectra in case of the vein, it can be concluded that the $\mu_{\mathrm{eff}}$ spectrum given in Fig. 5 for the tissue segment between the irradiation points at the forearm surface and the vein is not trustworthy. A possible reason for the unsuccessful spectral correction for the vein is the fact that the tissue segment that is optically characterized using the MIS method, i.e., the segment between the irradiation points and the vessel, is located in a shallower tissue region than in the case of the artery. For the accuracy of the MIS method, the structure of the tissue that lies between the source and the vessel is crucial. In particular, MIS relies on the assumption that there exist prevalent tissue regions between the irradiation positions and the vessel that can be modeled as an optically quasihomogeneous medium in which the fluence can be described by the analytical diffusion approximation for a semi-infinite medium. This assumption seems to be more violated in case of the vein, which is plausible, on the one hand, as the photon paths are influenced more strongly by superficial tissue layers, which are likely to have more pronounced optical inhomogeneities, and on the other hand, due to the presumably stronger influence of the boundary (skin surface). It should be noted that we did not comment on the accuracy of the $\mu_{\mathrm{eff}}$ spectra earlier, as neither a reference for the optical properties is available nor had the performance of the MIS method been validated *in vivo*. The metric analysis proposed in this paper—or any method that allows one to assess the trustworthiness of spectral fits—could be a promising approach to systematically investigate the range of applicability and validity of the MIS method; however, this was not within the scope of this study.

When comparing Figs. 4(a), 4(b) and 6(a), 6(b) with the blood absorption spectra shown in Fig. 3(a), it can be observed that the OA spectra are more similar to actual blood spectra before spectral correction than after correction. However, as mentioned in Sec. 3.1, this observation is not meaningful, as in this tissue depth a substantial spectral distortion is expected and the uncorrected OA spectra therefore in general do not reflect the absorption spectra of the blood in the vessels. Ideally, it would be expected that a spectral correction brings the trends of the OA spectra closer to the spectral trends of the blood in the vessels. However, errors in the estimated $\mu_{\mathrm{eff}}$ spectra propagate into the corrected OA spectra, and in case of the vein, errors in $\mu_{\mathrm{eff}}$ led to a dissimilarity between the corrected OA spectra’s trends and the spectral trend of actual blood. We would like to underline that it is exactly the goal of the proposed metric to identify such cases. In particular, the *in vivo* results presented in this section highlight what has been mentioned in Sec. 2.3, namely that it is not sufficient to rely on the physiological plausibility of ${\mathrm{SO}}_{2}$ values retrieved from spectral fits. We would like to emphasize again that the reliability assessment based on our metric is independent of the technique employed for spectral correction.

The central message of this paper is that in quantitative OA imaging, an assessment of the reliability of spectral fits is advisable, and we suggest that quantifying the similarity between trends of OA spectra and trends of fitted blood spectra constitutes a valuable approach. To showcase this, we proposed a metric on which this similarity analysis can be based. The choice of the particular metric was based on an important observation: in Figs. 3(a) and 3(c), it could be seen that the ${\mathrm{SO}}_{2}$ is related to the slopes of the blood spectrum left and right of the isosbestic point in that the slope pairs ($m_{1}$, $m_{2}$) corresponding to actual blood are uniquely linked to the ${\mathrm{SO}}_{2}$ [see reference line in Fig. 3(c)]. One can think of a spectral fit as the characterization of the trend of a blood spectrum with only one free parameter (the ${\mathrm{SO}}_{2}$), whereas the metric characterizes the trend of a spectrum in two dimensions ($m_{1}$ and $m_{2}$), thus providing a more detailed description of the trend. In general, a measurement outcome can only be deemed reliable, if alternative methods yield similar results. In our case, we compare the trend that is given by ($m_{1}$, $m_{2}$) with the trend suggested by the ${\mathrm{SO}}_{2}$ resulting from the spectral fit. The two methods are in agreement, if the cluster in the metric plot is located close to the points on the reference line covering the same color range. The proposed metric is sensitive to systematic biases between OA spectra and fitted spectra. The type of bias the metric detects (dissimilarities in the slopes of the spectra left and right of the isosbestic point) is highly relevant with regard to the ${\mathrm{SO}}_{2}$ since, as mentioned above, the ${\mathrm{SO}}_{2}$ level and the slopes are strongly correlated. Yet, it is important to mention that our metric is very likely not the only metric suitable for the purpose, i.e., for quantifying the similarity between measured and fitted spectra. Another potential approach to quantify the similarity would, e.g., be to apply principle component analysis to both the OA spectra and the fitted blood spectra. It was, however, beyond the scope of this paper to perform a detailed study on the optimal choice of the metric.

## 4 Conclusion

A central challenge in *in vivo* quantitative OA imaging is the validation of the ${\mathrm{SO}}_{2}$ values retrieved in vessels, following the optical characterization of the tissue and the spectral correction of the measured OA spectra that were initially distorted due to the wavelength-dependent optical attenuation of light. Throughout this paper, we have shown that one valuable piece of information that is directly available from the OA measurements is the trends of the OA spectra in the vessel supports, and not only after the spectral correction but also before. The intention is, before spectral correction, to identify systematic trends of spectra in the supports and, after correction, to assess the similarity between the trends of the corrected OA spectra and those of the fitted blood spectra. This two-step analysis can be extremely helpful to distinguish errors in the measurements and/or image analysis (e.g., imperfect support segmentation, influence of motion artifacts, or a low SNR) from an erroneous spectral correction (i.e., incorrect optical characterization of the tissue). With the examples of *in vivo* measurements from a human artery and vein, we have demonstrated that the analysis of the trends is essential: retrieving physiologically sound ${\mathrm{SO}}_{2}$ values does not necessarily indicate that the spectral correction has been successful. In particular, the OA measurements acquired from the vein illustrate a case in which the ${\mathrm{SO}}_{2}$ retrieved after correction could misleadingly indicate a successful correction and the metric can be used to disprove this, by showing that the trends of the corrected OA spectra do not agree with the trends of the fitted blood spectra.

The main focus of this paper was to draw attention to the fact that analyzing the similarity between the OA spectra and the fitted blood spectra constitutes an easy way of assessing the reliability of spectral fits. The metric used in this study to quantify spectral trends was chosen with the goal to provide a representative similarity measure that is relevant for the ${\mathrm{SO}}_{2}$. It can easily be incorporated into existing quantitative OA approaches since the analysis is computationally very lightweight and does not need *a priori* knowledge. With the corresponding metric plots, we have introduced a concise representation of the results. These metric plots contain information across the supports on (i) the uniformity of the trends of the OA spectra (this could, in principle, be adapted to perform an automated segmentation of the OA images), (ii) the fitted ${\mathrm{SO}}_{2}$ levels, and (iii) the similarity between the OA spectra and the fitted blood spectra. In recent years, promising developments have taken place in the field of quantitative OA imaging (see, e.g., Refs. 44–47) and our metric analysis complements these so as to move forwards with the generation of more reliable medical images.
