# Supplementary material for: Reliability assessment for blood oxygen saturation levels measured with optoacoustic imaging
Source: J Biomed Opt. 2020 Apr 22;25(4):046005. doi: 10.1117/1.JBO.25.4.046005 (PMC7175414; doi:10.1117/1.JBO.25.4.046005)
Supplement: Supplementary file 1 [file JBO_025_046005_SD001.pdf]

Supplementary Material: Reliability assessment for blood oxygen saturation levels measured with optoacoustic imaging

Leonie Ulrich[a], **Kai Gerrit Held**[a,b], **Michael Jaeger**[a], **Martin Frenz**[a,*], **Hidayet Günhan Akarçay**[a]
[a]University of Bern, Institute of Applied Physics, Biomedical Photonics, Bern, Switzerland
[b]ABB Switzerland, Corporate Research, Baden-Daettwil, Switzerland

*Address all correspondence to Martin Frenz, E-mail: martin.frenz@iap.unibe.ch

Multiple Irradiation Sensing for Optical Tissue Characterization

Principle

For optical characterization of the tissue surrounding the vessels we used multiple irradiation sensing (MIS), a method where blood vessels are used as intrinsic fluence detectors that probe the tissue from within. This concept was originally introduced by Zemp *et al.* (see Refs. 21,22 in the main text). It has been extended in our group to perform spectral analysis by using multiple wavelengths (Refs. 23,24) and has recently been developed towards real-time application by Jeng *et al.* and Kim *et al.* (Refs. 48,49). Here, the MIS method is, to the best of our knowledge, applied for the first time *in vivo*. Optoacoustic (OA) signals emanating from the vessels, which are proportional to the absorbed energy density in the vessels, are recorded for different irradiation positions and a light propagation model is then employed to retrieve the effective attenuation coefficient μ_{eff} of the tissue. We use a semi-empirical model (introduced in Ref. 23) which is based on the assumption that there exist prevalent tissue regions between the illumination positions and the vessels which can be modeled as an optically quasihomogeneous medium, *i.e.*, the photon paths between the irradiation spots and the detectors (the vessels) are not obstructed by absorbing structures or affected by boundaries. In these regions, the fluence Φ can be described by the analytical diffusion approximation for a semi-infinite medium (Refs. 23,24):

$$\Phi(\mathbf{r}) \propto \frac{z}{d^3}[1+\mu_{\text{eff}}\cdot d]e^{-\mu_{\text{eff}}\cdot d}\,. \tag{1}$$

Here, z is the depth of the detection point $\mathbf{r}$ and d is the distance between the detection point and the illumination source.

For the MIS analysis in this study, the detection point $\mathbf{r}$ can be identified with the position (x^c_{ij}, z^c_{ij}) of the vessel, see Sec. 2.2 of the main text. Following this nomenclature, the depth equals z^c_{ij}, for irradiation position x_i and wavelength λ_j, and the distance between detection point and illumination source is $d_{ij} = \sqrt{(x^c_{ij}-x_i)^2+(y_0)^2+(z^c_{ij})^2}$. For our analysis, we neglected variations in the vessel position within a data set acquired at the same wavelength (*i.e.*, assumed the position of the vessel center to be (x^c_{1j}, z^c_{1j}) for all irradiation positions), since they were observed to be much smaller than variations in position between different wavelengths.

For every irradiation position x_i and wavelength λ_j, we determined the mean amplitude $\langle S_{ij}\rangle$ of the vessel's OA signal by averaging the pixel values in the OA image $S_{ij}(\mathbf{r})$ over the respective support. In case of a homogeneous medium, it would be sufficient to perform a single fit of the

analytical fluence model (Eq. (1)) to the amplitudes $\langle S_{ij} \rangle$ determined for different d_{ij} in order to retrieve μ_{eff} (see Ref. 24). However, the envisaged purpose of the semi-empirical model was, as described in Ref. 23, the optical characterization of tissue in situations where the analytical diffusion approximation for a semi-infinite medium fails at accurately modeling the fluence. In particular, the model was designed to account for the fact that the diffusion approximation is expected to break down when the light propagation is influenced by boundaries or optical heterogeneities/by shadowing due to absorbing structures. Therefore, instead of simply fitting Eq. (1) to all $\langle S_{ij} \rangle$, a different procedure was carried out: The slope m_{ijk} of $\log(\langle S_{ij} \rangle d_{ij}^3)$ was determined for every d_{ij}, by fitting a first-order polynomial to the data points around d_{ij}, taking into account k points in total. This procedure was repeated for different k ranging from 2 to N, the total number of irradiation positions. Illustrations of the segmented OA signal and the fitting procedure are shown in Fig. 1 (a) and (b), respectively. For each slope m_{ijk}, $\mu_{\text{eff},ijk}$ was calculated by inverting the first-order term m' of the Taylor expansion of the diffusion approximation[1]

$$m' = \frac{\partial \log(\Phi \cdot d^3)}{\partial d} = -\mu_{\text{eff}} + \frac{\mu_{\text{eff}}}{1 + \mu_{\text{eff}} \cdot d} \tag{2}$$

$$\Rightarrow \mu_{\text{eff}} = -\frac{m' \cdot d - \sqrt{m'^2 \cdot d^2 - 4 \cdot m' \cdot d}}{2 \cdot d} \tag{3}$$

and setting m' to m_{ijk}, and d to the center of the d_{ij} of the k data points taken into account for the respective fit. We then performed a statistical analysis of $\mu_{\text{eff},ijk}$ realizations, assuming that the most frequently occurring effective attenuation coefficient optically characterizes a quasihomogeneous tissue segment. For each wavelength λ_j, we histogrammed all $\mu_{\text{eff},ijk}$ values and determined $\mu_{\text{eff},j}$ of the tissue segment as the peak position of a Gaussian fitted to the histogrammed $\mu_{\text{eff},ijk}$ (see inset in Fig. 1 (b)). The standard deviation of the Gaussian was taken as a measure for the uncertainty of $\mu_{\text{eff},j}$. In comparison to the method described in Ref. 23, the analysis has been extended by an additional step: we identified the set of irradiation positions corresponding to a tissue segment for which the diffusion approximation is valid. An irradiation position x_i was considered to belong to this set if the absolute difference between $\mu_{\text{eff},j}$ and the $\mu_{\text{eff},ijk}$ was smaller than the uncertainty of $\mu_{\text{eff},j}$ for all wavelengths λ_j. Spectral correction was performed only for these irradiation positions. With $\mu_{\text{eff},j}$, we analytically calculated the fluences $\Phi_{ij}(\mathbf{r})$ for all λ_j and the identified x_i, based on the diffusion approximation for semi-infinite media (Eq. (1)), with the pixel resolution of the OA images, and performed a spectral correction by pixelwise dividing the OA image $S_{ij}(\mathbf{r})$ by the corresponding fluence $\Phi_{ij}(\mathbf{r})$.

Results

Figure 2 (a) and (b) display histograms of realizations of $\mu_{\text{eff},ijk}$ for all d_{ij} and window sizes k, together with the best fits of Gaussian distributions to the data, for both the artery and the vein. It was observed that the influence of the choice of bin size for the histograms on the peak positions of the Gaussians is negligible compared to their widths. The spectra of the effective attenuation coefficient $\mu_{\text{eff},j}$ are given in Fig. 2 (c), for the tissue segments between the irradiation positions on the forearm surface and the artery and the vein, respectively. It can be seen that both the absolute

[1] Even though the calculations in Ref. 23 were done using the correct formula, we have noticed an unfortunate error in Eq. (2) of the publication. The correct formula for m' is given here.

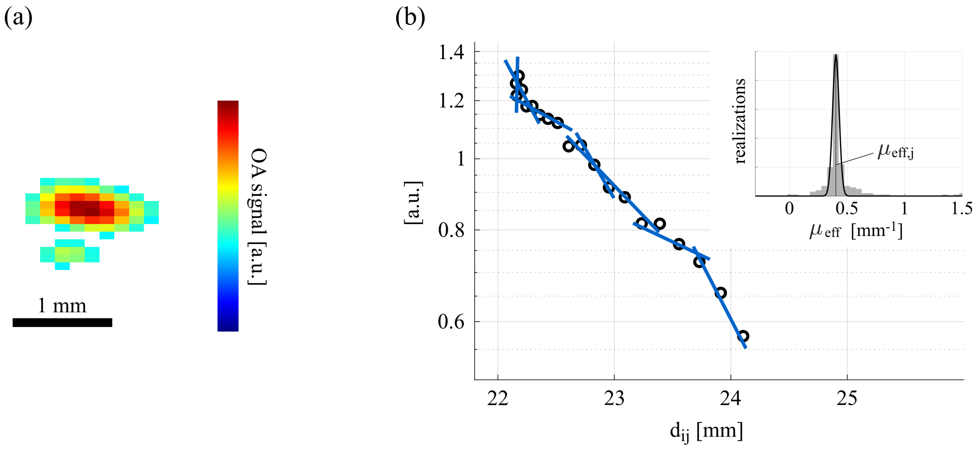

Fig 1 (a) Example of the OA signal in a segmented support, for one irradiation position x_i and wavelength λ_j. $\langle S_{ij} \rangle$ is the average of all pixel values across the support. (b) Illustration of $\langle S_{ij} \rangle d_{ij}^3$ as a function of d_{ij} (black circles, plotted logarithmically). For all wavelengths λ_j, slopes are fitted for varying d_{ij} and varying window sizes k (blue lines). $\mu_{\text{eff},j}$ are the peak positions of Gaussians fitted to histogrammed $\mu_{\text{eff},ijk}$ realizations (see inset).

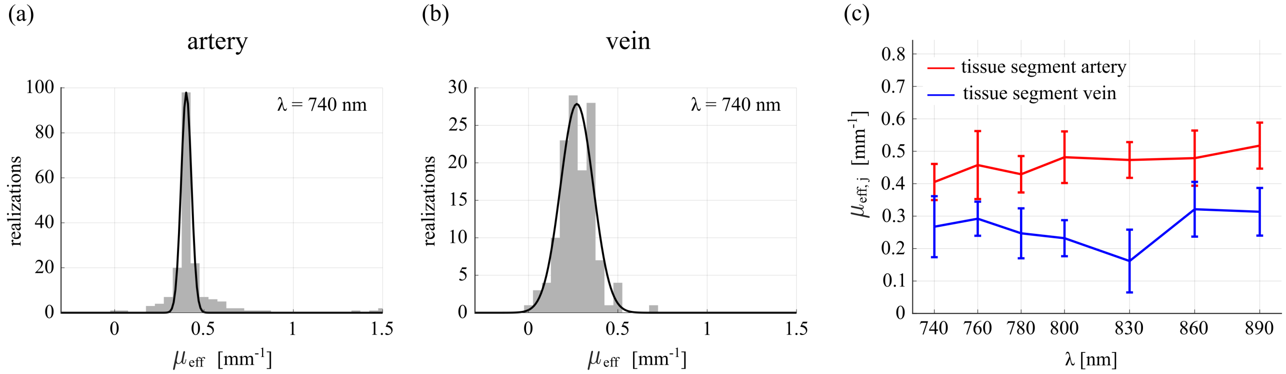

Fig 2 (a), (b): Histogram of realizations of $\mu_{\text{eff},ijk}$ values (compare Fig. 1 (b)), together with the Gaussian fit (black line), for the artery and the vein, respectively. The peak position of the Gaussian determines $\mu_{\text{eff},j}$. (c) μ_{eff} spectra estimated for the tissue segments between the forearm surface and the artery (red) and the vein (blue), error bars represent the standard deviations of the Gaussian distributions fitted to the histograms. A video showing the figure parts (a) and (b) for all λ_j is available (Video S1 MPEG, 5.3 MB).

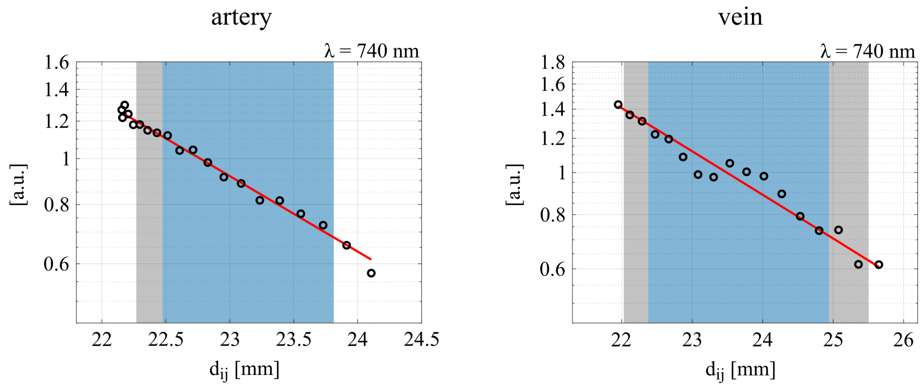

Fig 3 $\langle S_{ij}\rangle d_{ij}^3$ as a function of the source-detector distance d_{ij} (black circles), together with $\Phi_{ij}d_{ij}^3$ (red line), shown in a logarithmic plot, for the artery (left) and the vein (right). For better visual comparison, $\langle S_{ij}\rangle d_{ij}^3$ and $\Phi_{ij}d_{ij}^3$ were normalized to their respective mean value. Note that, for the vein, we observed a lower signal-to-noise ratio than for the artery and thus discarded data points for $i \geq 18$. The gray band corresponds to the set of irradiation positions for which the absolute difference between $\mu_{\text{eff},j}$ and the $\mu_{\text{eff},ijk}$ was smaller than the uncertainty of $\mu_{\text{eff},j}$ for the respective wavelength. The overlap of the sets identified for all wavelengths, defining the optically quasihomogeneous tissue region, is indicated in blue. A video showing the figure for all λ_j is available (Video S2, MPEG, 5.6 MB).

values of $\mu_{\text{eff},j}$ as well as the trends of the spectra differ between the two vessels. These differences could possibly be explained by the fact that the tissue segment probed when analyzing signals stemming from the artery has different optical properties than that probed in the case of the vein.

Figure 3 illustrates the data points $\langle S_{ij}\rangle d_{ij}^3$ together with $\Phi_{ij}d_{ij}^3$ (where Φ_{ij} denotes the fluence at the position of the vessel) and visualizes the range of source-detector distances d_{ij} defining the optically quasihomogeneous tissue region where the light propagation can be described based on Eq. (1), for both the artery and the vein. For the artery, the measured OA data and the fluence model agree very well for the identified region, whereas the agreement is lower for the vein, presumably, as mentioned in the main text, due to a stronger influence of the boundary (skin surface) and the stronger inhomogeneity of the probed tissue segment. The very good agreement for the artery confirms our hypothesis: despite the presence of heterogeneities in the forearm, there exist tissue regions for which the simple analytical diffusion approximation can be used to model the light propagation.
